# Effectiveness of sublingual immunotherapy with house dust mite drops in asthmatic children at different ages

**DOI:** 10.3389/fped.2023.1170860

**Published:** 2023-06-12

**Authors:** Tao Ai, Peilin Zhang, Ronghua Luo, Yinghong Fan, Wanmin Xia, Li Wang

**Affiliations:** Pediatric Respiratory Medicine Department, Chengdu Women’s and Children’s Central Hospital, School of Medicine, University of Electronic Science and Technology of China, Chengdu, China

**Keywords:** sublingual immunotherapy, asthma, children, pulmonary function, age difference

## Abstract

**Background:**

Respiratory allergies in children, such as asthma and rhinitis, are becoming progressively common every year. Recent studies found that pediatric patients with asthma receiving regular medication and specific immunotherapy (SIT) had improved therapeutic outcomes in a wide age range. However, there are few studies that have examined the effectiveness of SIT treatment in children with allergic asthma at different ages in terms of the degree of asthma control, improvements in lung function, and changes in exhaled nitric oxide (FeNO).

**Method:**

A total of 200 asthmatic pediatric patients who had been receiving regular treatment for at least a year were split into the observation and the control groups, which depended on whether sublingual immunotherapy was added based on conventional treatment medicines. The children who were divided by an age cut-off of 6 years old in these two groups were compared before and after therapy based on the exhaled levels of FeNO, pulmonary function, visual analog scale, medication scores, daytime and nighttime ratings of asthma symptom, and rhinitis symptom scores.

**Results:**

Before treatment, there was no significant difference between the observation group and the control group in various indicators of the patients under 6 years old; and in the older children (6–16 years old) group, the scores of FVC, FEV1, and FEF25 in the observation group were significantly lower than those in the control group (*P* < 0.05). The FEF75, FEF50, FEF25, and MMEF75/MMEF25 indexes in the observation group were significantly higher than those in the control group after treatment (*P* < 0.05), but there was no statistical significance in other indexes (*P* > 0.05). The scores of ACT, FEF75, FEF50, MMEF72/MMEF25, and FeNO in the observation group were all higher than those in the control group after treatment (*P* < 0.05), and the differences in other indexes were not statistically significant (*P* > 0.05). Between the young-age group and the elder group, there was no significant difference in all indexes in the observation group before and after treatment (*P* > 0.05).

**Conclusion:**

Children with asthma of all ages can considerably benefit from sublingual immunotherapy. Specifically, younger patients showed greater tendency on the improvement of small airway resistance, whereas school-age children with asthma significantly improved their small airway resistance as well as their asthma control and inflammation alleviation.

## 1. Introduction

Allergen-specific immunotherapy (ASIT), also allergen desensitization therapy, is introduced by Dr. Leonard Noon and Dr. John Freeman for managing IgE-mediated allergic diseases in 1911, which utilizes allergen to redirect inappropriate immune responses to build up the host's tolerances and diminish symptoms by administrating allergen extract at escalating doses over a period of 3–5 years (1–4). For almost a century, ASIT has been developed into several administration pathways, such as subcutaneous immunotherapy (SCIT), sublingual immunotherapy (SLIT), bronchial immunotherapy (BIT), oral immunotherapy (OIT), local nasal immunotherapy (LNIT), epicutaneous immunotherapy (EPIT), and intralymphatic immunotherapy (ILIT) (5). Among them, SLIT, as a non-invasive procedure, is raising a lot of interest and popularity worldwide, because of its significant therapeutic outcome, reduced side effects, outstanding stability, and being user-friendly (5, 6). The dose of allergen that is exposed to the body during specific immunotherapy is significantly higher than that of daily exposure. The initial phase of dose escalation promoted the transformation of Th0 cells into the regulatory T lymphocytes (Treg), promoted the production of IL-10 and transforming growth factor (TGF)-β, and inhibited the Th2-cell-related immune response. As the dose of the allergen intake increases to the maintenance dose, it will trigger a switch in the direction of the Th0 to Th1 cells. At the same time, immunotherapy induced the B lymphocytes to produce blocking IgG4 antibodies and reduce IgE antibody production. After the allergen is introduced into the body, IgG4 antibodies competitively bind to it before triggering a cascade of anaphylaxis, which inhibits and downregulates the allergen-induced sequence of immune responses. Through this immune mechanism, the immune tolerance to allergens is gradually promoted.

The therapeutic efficacy and safety of SLIT is not only confirmed by the World Health Organization (WHO) and the World Allergy Organization (WAO), but they also recommend SLIT as an initial treatment for both adult and pediatric allergic diseases. SLIT has emerged as an effective and safer way. In view of the convenience of self-administration in the patients’ home, decreased risks of side effects, painless treatment, and unnecessary need for frequent physician attendances, SLIT outstands itself in pediatric ASIT from preschool to teenagers with allergic rhinitis (AR), allergic asthma (AS), atopic dermatitis (AD), allergic conjunctivitis (AC), and other allergic diseases (7–9). Even though the clinical efficacy of SLIT in children has been established, the research on its clinical efficacy and safety in patients at different ages remains rare. Therefore, we compared pulmonary function and symptom control scores before and after SLIT to evaluate whether therapeutic efficacy possesses age difference.

## 2. Method

### 2.1. Patients

From February 2019 to January 2021, 200 children with asthma admitted in our hospital, whose skin prick tests indicated positive results for dust mites, were divided into two groups randomly: (1) Observation group (100 cases): children treated with sublingual dust mite drops, at the same time, according to the condition to use symptomatic treatment drugs for asthma. (2) Control group (100 cases): children only use symptomatic treatment drugs for asthma. The children in the observation and control group were divided into the young-age group (<6 years old) and the elder group (≥6 years old), incorporating other allergens (such as milk, eggs, beef, fish and shrimp, mold, and pollen) except dust mites. The study was approved by the Ethics Board of the Chengdu Women and Children Center Hospital, and an informed consent to participate in the study was provided by the parents of the patients.

The inclusion principles included the following: (1) patients were diagnosed and treated according to the standardized diagnosis and treatment recommendations of childhood bronchial asthma (2020) (10); (2) all children were tested for allergens before treatment, and dust mites were positive, with or without other allergens; and (3) children with asthma in the remission stage.

The exclusion criteria included the following: (1) acute asthma (FEV1 less than 70% of the predicted value); (2) severe allergic reaction; and (3) withdrawal from the study or loss of follow-up due to various reasons.

C-ACT, visual analog scale (VAS) score, drug score, lung function, and exhaled nitric oxide were evaluated once before enrollment. The detailed description for the patients’ treatment, pulmonary function, determination of nitric oxide, and evaluation indicators assessment has been published before (11).

## 3. Results

### 3.1. Basic characteristics

After a year of follow-up, 160 out of the 200 patients were analyzed, of which 8 patients terminated desensitization because of acute attack of asthma following an acute lower respiratory tract infection (FEV1 less than the 70% expected value), 14 patients were lost to follow-up, and 18 voluntarily withdrew. Finally, there were 71 patients in the observation group and 89 patients in the control group who finished the study, while there is no significant difference between the observation group (71 patients) and the control group (89 patients) in terms of gender (observation group: male 66.2%, control group: male 57.3%, *P* = 0.251) and age (observation group: 7.2 ± 2.9 years old, control group: 7.0 ± 2.3 years old, *P* = 0.714). Also, we divided the patients at the age cut-off of 6 years old, resulting in 59 patients of under 6 years old and 101 patients of older than 6 years old, while there is no significant difference between the two groups in terms of gender (<6-year-old group: male 57.6%, ≥6-year-old group: male 63.4%, *P* = 0.472). Specifically, there are 26 patients under 6 years old in the observation group, defined as the young-age group, while there are 45 children of over 6 years old, defined as the elder group. The gender difference in these two groups is also not significant (young-age group: male 73.1%, elder group: male 62.2%, *P* = 0.352).

### 3.2. Indexes comparison between the observation group and control group in the young-age group and elder group before treatment

Before treatment, between the observation group and control group, we previously found that FVC, FEV1, and PEF25 scores in the observation group was significantly lower than that in the control group (*P* < 0.05), while all other indexes did not show significant differences. In this study, we found that all indexes in the patients under the age of 6 years did not exhibit a significant difference between the observation group and control group, while FVC, FEV1, and FEF25 scores in the observed patients over 6 years old were found significantly lower than that in the control group (*P* < 0.05) (Table 1).

**Table 1:** Indexes comparison between the observation group and control group in the young-age group and in the elder group before treatment.

Indexes	Young-age group				Elder group			
	Observation group	Control group	*Z/t*	*P*	Observation group	Control group	*Z/t*	*P*
	*n* = 26	*n* = 33			*n* = 45	*n* = 56		
ACT	14.5 (11.75–15)	16 (12–19)	1.494	0.135	13 (10–17)	16 (10–19)	1.642	0.101
VAS	3.5 (3.5–25)	3 (1–5)	1.247	0.212	4 (2–6)	3 (1–6)	1.49	0.136
FVC	90.00 ± 12.33	96.86 ± 16.94	1.732	0.089^a^	90.3 (83.95–95.4)	98.2 (87.9–104.6)	3.239	0.001*
FEV1	91.27 ± 12.21	97.22 ± 18.09	1.438	0.156^a^	88.7 (81.5–96.45)	95.3 (86.3–104.0)	3.126	0.002*
FEF	92.40 (87.58–97.10)	95.20 (82.10–104.10)	0.435	0.663	91.61 ± 14.97	96.53 ± 17.02	1.523	0.131^a^
FEF75	61.20 (41.85–76.13)	52.50 (43.35–61.25)	0.977	0.328	54.8 (42.1–68.35)	48.85 (40.18–63.98)	0.929	0.353
FEF50	68.75 (56.68–84.35)	64.20 (56.15–86.05)	0.099	0.921	66.76 ± 16.05	67.49 ± 20.7	0.194	0.846^a^
FEF25	70.45 (60.58–81.15)	74.70 (63.8–98.00)	1.412	0.158	65.3 (49.2–75.55)	82.6 (66.58–94.7)	3.837	<0.001*
MMEF75/MMEF25	68.45 (54.55–83.40)	66.10 (55.50–81.80)	0.107	0.915	61.8244 ± 16.40	65.99 ± 20.29	1.116	0.269^a^
FeNO	29.00 (23.00–48.25)	30.00 (12.50–55.50)	1.092	0.275	33.00 (26.00–45.00)	35.00 (18.00–56.00)	0.396	0.692

VAS, visual analog scale; FeNO, nitric oxide.

^a^ Student'*t*-test. Mann–Whitney *U*-test is used for the rest.

* *P* < 0.05.

### 3.3. Indexes comparison between the observation group and control group in the young-age group and elder group after treatment

After treatment in the young-age group, there were significant differences in FEF75 (*z* = 3.512, *P* < 0.001), FEF50 (*z* = 3.535, *P* < 0.001), FEF25 (*z* = 2.794, *P* = 0.005), and MMEF (*z* = 3.061, *P* = 0.002) between the observation group and the control group, which were also higher in the observation group. In the elder group, ACT (*z* = 2.216, *P* = 0.027), FEF75 (*t* = 5.324, *P* < 0.001), FEF50 (*z* = 2.993, *P* = 0.003), and MMEF (*t* = 2.021, *P* = 0.046) were found higher in the observation group, and the difference was statistically significant. Moreover, the significant difference in FeNO was found between the observation group and the control group, and the difference was larger in the older children than in the younger children (*z* = 2.378, *P* = 0.017) (Table 2).

**Table 2:** Indexes comparison between the observation group and control group in the young-age group and in the elder group after treatment.

Indexes	Young-age group				Elder group			
	Observation group	Control group	*Z/t*	*P*	Observation group	Control group	*Z/t*	*P*
	n = 26	n = 33			n = 45	n = 56		
ACT	24.5 (20–27)	23 (19–27)	0.737	0.461	26 (20–27)	21 (19–27)	2.216	0.027*
VAS	0 (0–1)	0 (0–1)	4.81	0.63	0 (0–1)	1 (0–1)	1.551	0.121
FVC	105.84 ± 9.67	105.03 ± 13.50	0.26	0.796^a^	101.1 (94.8–109.9)	106.4 (97.43–111.65)	0.892	0.373
FEV1	108.95 (99.45–114.18)	105.60 (98.9–110.00)	1.321	0.187	102.2 (94.3–112.65)	103.6 (98.3–112)	0.400	0.689
PEF	108.48 ± 9.16	106.48 ± 15.65	0.579	0.565^a^	104.84 ± 14.68	107.97 ± 15.45	1.032	0.305^a^
FEF75	92.35 (75.4–109.35)	70.20 (62.65–76.35)	3.512	<0.001*	90.47 ± 23.35	68.05 ± 17.74	5.324	<0.001^a^^,^*
FEF50	97.20 (83.35–106.95)	76.8 (69.7–86.7)	3.535	<0.001*	92.4 (76–105.95)	78.9 (67.43–87.3)	2.993	0.003*
FEF25	101.35 (88.6–107.55)	89 (74.25–98.30)	2.794	0.005*	97.6 (81.2–106.75)	89.1576.75–102.5)	1.476	0.14
MMEF75/MMEF25	97.10 (87.43–109.65)	78 (69.35–88.65)	3.061	0.002*	88.04 ± 20.90	80.21 ± 18.00	2.021	0.046^a^^,^*
FeNO	15.50 (10.00–27.25)	16.00 (13.00–33.00)	0.558	0.577	16.00 (12.00–24.00)	20.00 (15.00–33.50)	2.378	0.017*

VAS, visual analog scale; FeNO, nitric oxide.

^a^ Student'*t*-test. Mann–Whitney *U*-test is used for the rest.

* *P* < 0.05.

### 3.4. Indexes comparison between the young-age and elder patients in the observation group before and after treatment

Between the young-age group and elder group, there was no significant difference in all indexes in the observation group before and after treatment (*P* > 0.05) (Table 3).

**Table 3:** Indexes comparison between the young-age group and elder group before and after treatment in the observation group.

Indexes	Before treatment				After treatment			
	Young-age group	Elder group	*Z/t*	*P*	Young-age group	Elder group	*Z/t*	*P*
	*n *= 26	*n *= 45			*n *= 26	*n *= 45		
ACT	14.50 (11.75–15.00)	13.00 (10.00–17.00)	0.541	0.588	24.50 (20.00–27.00)	26.00 (20.00–27.00)	0.416	0.677
VAS	3.5 (3–5.25)	4 (2–6)	0.446	0.656	0.00 (0–1)	0 (0–1)	0.43	0.667
FVC	87.6 (80.00–99.25)	90.3 (83.95–95.40)	0.173	0.863	105.84 ± 9.67	102.99 ± 13.33	0.133	0.343^a^
FEV	91.0 (79.88–100.88)	88.70 (81.50–96.45)	1.307	0.191	108.37 ± 12.13	104.30 ± 13.10	1.296	0.199^a^
FEF	92.52 ± 9.32	91.61 ± 14.97	0.318	0.752^a^	108.48 ± 9.15	104.84 ± 14.68	1.285	0.203^a^
FEF75	59.51 ± 22.19	55.58 ± 17.05	0.838	0.405^a^	90.21 ± 26.06	90.47 ± 23.35	0.043	0.966^a^
FEF50	68.62 ± 14.96	66.76 ± 16.05	0.48	0.633^a^	95.90 ± 21.16	90.98 ± 19.41	0.996	0.323^a^
FEF25	70.45 (60.58–81.15)	65.30 (49.20–75.55)	1.826	0.068	101.35 (88.60–107.55)	97.60 (81.20–106.75)	1.021	0.307
MMEF75/MMEF25	68.28 ± 15.89	61.82 ± 16.40	1.617	0.110^a^	97.10 (87.43–109.65)	91.70 (69.65–101.40)	1.414	0.157
FeNO	29.00 (23.00–48.25)	33.00 (26.00–45.00)	0.836	0.403	15.50 (10.00–27.25)	16.00 (12.00–24.00)	0.078	0.938

VAS, visual analog scale; FeNO, nitric oxide.

^a^ Student'*t*-test. Mann–Whitney *U*-test is used for the rest.

## 4. Discussion

Bronchial asthma is a common chronic airway inflammatory disease in children. Atopy status is one of the most common risk factors for bronchial asthma in children, especially for children with combined allergic rhinitis or atopic dermatitis (12). The role of the host response to asthma pathophysiology is important, and immunotherapy targeting allergens can be used as a complement to asthma treatment. Dust mites are the most common allergen-triggering factor for asthma attacks worldwide, and immunotherapy against dust mites has developed as one of the important treatment for asthma management, which mainly involved SCIT and house dust mite SLIT (13). SCIT is now used sparingly due to its safety and compliance advantages in children (13). SLIT induces the activation of Treg and Breg cells, modulates allergen-specific IgE and IgG antibody-mediated immune responses, and inhibits mast cell and basophil degranulation, thus acting to reduce allergic inflammation (14). Mohammad et al. compared the correlation between perfected skin prick tests, serum IgE testing, and asthma occurrence at age 3, 5, 8, and 11 years and found out that the positive relationship between positive skin prick tests and asthma incidence increased with age, while the correlation between elevated serum IgE and asthma incidence may decrease (15). Therefore, the efficacy of sublingual immunotherapy for asthma in children of different ages remains to be proven.

Symptomatic drugs for asthma mainly include regular inhaled corticosteroids in low dose and on-demand inhaled beta 2-agonist. In this research, all the patients in the control group received symptomatic drugs, and in the observation group, either young-age or elder children, all accepted SLIT and symptomatic drugs for asthma. The only difference is that the young-age group used pressurized metered dose inhaler with a spacer, but the elder group used dry powder inhaler. Studies have shown that the two types of inhalation administration are equally effective in children with asthma (16, 17).

Symptom control is an important reference for asthma control, and ACT provides a better picture of symptom control in children with asthma (12). In several meta-analyses, better asthma symptom scores were found in the sublingual immunotherapy group compared with the placebo group, but the credibility of the results was reduced by high heterogeneity in the included studies or limited sample size (18–20). Our previous study found that combination therapy of SLIT and regular treatment can significantly improve ACT scores in children with asthma (11). To have a better understanding on whether age difference would influence the therapeutic outcome, we divided the patients at age cut-off of 6 years old, which are the young-age group (<6 years old) and the elder group (≥6 years old). We found that in the elder group, the combined therapy improved ACT scores in children, while there is no significant advantage of SLIT in ACT in the observed young-age group compared with the control group. This suggests that SLIT is more effective in children aged 6 years and older in terms of asthma symptom control.

Pulmonary function tests also play an essential role in the diagnosis and follow-up of asthma patients (12). Asamoah et al. found that SLIT improved patients’ medication and symptom scores and measures of bronchial hyper-reactivity, but pulmonary function improvement was unclear through a systematic review of studies in nine databases (21). In contrast, another systematic review on the efficacy of desensitization in children with asthma noted low strength evidence that desensitization improved FEV1 in children with asthma (22). In addition, a meta-analysis found that subcutaneous desensitization did not contribute to PEF, FEV1, and FVC in asthmatic patients (23). Our previous study found that combined desensitization improved FVC, FEV1, FEF75, FEF50, FEF25, and MMEF75/MMEF25 more than the conventional asthma treatment group, with no difference in PEF improvement (24). In the present study, in children under the age of 6 years, FEF75, FEF50, FEF25, and MMEF75/MMEF25 in the observation group were seen to be more significantly improved than those in the control group. The above results in children aged 6 years and older were the same except for FEF25. This indicates that SLIT is more effective in small airway resistance in children of all ages.

FeNO can be used as an objective biomarker of eosinophilic airway inflammation, a non-invasive modality that has good value for the assessment of type II asthma. In a study of 112 adult patients with dust mite allergy with asthma, Hoshino et al. found that sublingual immunotherapy was effective in reducing the levels of FeNO (25). Similarly, by studying children aged 7–18 years suffering from allergic asthma and rhinitis, Djuric-Filipovic et al. concluded that sublingual immunotherapy combined with conventional anti-asthma treatment was more effective and had a better reduction in FeNO levels than the conventional anti-asthma treatment alone (26). The same conclusion was reached in our previous study (27). When we divided the study population into the younger group and the elder group, we found that patients over 6 years old showed an advantage in FeNO after SLIT treatment, suggesting that desensitization may be more helpful for airway inflammation levels in children with asthma older than 6 years.

In conclusion, we demonstrated that sublingual immunotherapy could improve the symptoms and pulmonary function in children with asthma, both in children under 6 years old and in children older than 6 years old. Interestingly, after comparison between the young-age and the elder groups, we found that SLIT may be more advantageous in children older than 6 years.

This research further indicates the clinical advantages of sublingual immunotherapy to anti-asthma treatment in children, especially for the use of young children to provide evidence-based medical evidence. But there are still some questions remained to be answered: (1) unlike the online single breath technique with constant expiratory flow used by the elder group, the patients in the young-age group mostly used the tidal breathing method to test FeNO, which may reduce the accuracy of FeNO testing for lower airway inflammation; (2) children with allergic rhinitis were not analyzed in this study as a subgroup, which may lead to bias.

## Data Availability

The raw data supporting the conclusions of this article will be made available by the authors, without undue reservation.
